# Safety of Early MRI Examinations in Severe TBI: A Test Battery for Proper Patient Selection

**DOI:** 10.3389/fneur.2020.00219

**Published:** 2020-04-07

**Authors:** Daniel Pinggera, Markus Luger, Iris Bürgler, Marlies Bauer, Claudius Thomé, Ondra Petr

**Affiliations:** ^1^Department of Neurosurgery, Medical University Innsbruck, Innsbruck, Austria; ^2^Department of Anesthesiology and Critical Care Medicine, Medical University Innsbruck, Innsbruck, Austria

**Keywords:** intrahospital transport, early magnetic resonance imaging, severe traumatic brain injury, intensive care management, critical ill patients

## Abstract

**Introduction:** Early magnetic resonance imaging (MRI) provides important information for management and prognosis in patients with severe traumatic brain injury (sTBI). Yet, optimal timing of MRI remains unknown. The aim of our study was to evaluate the safety of early MRI and to identify a method for appropriate patient selection to minimize adverse events related to the intrahospital transport (IHT) and the MRI examination.

**Methods:** Twenty-six patients with sTBI [mean Glasgow Coma Scale (GCS) 6, range 3–8] admitted to our neurosurgical ICU from 03/2015 to 12/2017 and receiving at least one MRI within the first 14 days after initial traumatic event were prospectively included in the study. The following requirements were fulfilled for at least 4 h prior to anticipated MRI: MAP > 70 mmHg, aPCO_2_ 30–40 mmHg, stable ICP < 25 mmHg. All relevant cardiopulmonary and cerebral parameters and medication were recorded. The following MRI sequences were performed: DWI, FLAIR, 3D T2-space, 3D T1 MPRAGE, 3D SWI, 3D TOF, pASL, and ^1^H/^31^P-MRS.

**Results:** Four females and 22 males (aged 23–78 years, mean 46.4 years) with a median GCS on admission of 5 (range 3–8) were analyzed. In total, 40 IHTs were performed within the first 14 days (mean 6 days, range 1–14 days). Mean pre-MRI ICP was 14.1 mmHg (range 3–32 mmHg). The mean post-MRI ICP was 14.3 mmHg (range 3–29 mmHg), decreasing to a mean ICP of 13.2 mmHg after 1 h (range 3–29 mmHg). There were no significant differences in ICP measurements before and after MRI (*p* = 0.30). MAP remained stable with no significant changes during the entire IHT and MRI. No other adverse events were observed as well.

**Conclusion:** Early MRI in acute severe TBI is feasible and safe. Yet, careful patient selection with prior adequate testing of cardiopulmonary and cerebral parameters is crucial to minimize transport- or examination-related morbidity.

## Introduction

Given the commonly known limitations of structural imaging methods in detecting clinically relevant, yet subtle, intracranial abnormalities after traumatic brain injury, magnetic resonance imaging (MRI) has become integral to neuroimaging diagnostics, especially in severe TBI. Early MRI can provide in-depth analysis of traumatic intracranial lesions, containing not only prognostic information but also potentially for therapeutic management, both with respect to short-term and long-term outcomes (1–4). Albeit early MRI may not dramatically alter initial patient management, it can be decisive for further treatment.

As patients with severe TBI are often on prolonged ventilation, early MRI is challenging as intrahospital transport (IHT) and the time in the scanner requires tedious monitoring. It is known that moving the patient from the NICU for therapeutic or diagnostic reasons is associated with numerous clinical issues such as increase in ICP, hemodynamic complications, or secondary insults (5–8). Especially for head-injured patients, IHT-related adverse events can lead to secondary brain damage, making a selection of possible candidates necessary (5, 9).

Importantly, both optimal timing of initial MRI in severe TBI and IHT-related complications due to early MRI continue to be debated. Thus, we prospectively analyzed 26 patients with severe TBI, who underwent at least one MRI within the first 14 days after the traumatic event, and therefore, an IHT was necessary. The aim of our study was to ensure safety for all patients and reduce adverse events. We established a test battery based on contemporary well-known neurointensive care standards in each patient prior to every anticipated MRI examination.

## Methods

### Patients

Consecutive patients between 18 and 85 years of age with severe TBI and an initial Glasgow Coma Scale (GCS) score of eight or less, who required treatment according to the BTF guidelines and underwent an MRI within the first 14 days after the trauma, were prospectively included in the study (10). All patients were monitored with an intraparenchymal ICP probe.

Exclusion criteria were contraindications to MRI (e.g., pacemaker, metal objects, etc.), and/or an unstable clinical condition disqualifying the patient to undergo an MRI examination safely. The study protocol was approved by the local ethics committee of the Medical University Innsbruck (AN2014-0201 339/4.6), and written informed consent was obtained from all included patients and/or from their legal guardians.

Twenty-six patients with severe TBI (mean GCS 6, range 3–8) admitted to our Department from March 2015 to December 2017 met the inclusion criteria and were prospectively included in the study.

### Test Battery

In order to ensure the safety of MRI scans, all patients were examined prior to anticipated MRI, needing to meet the following requirements:

- Mean arterial pressure above 70 mmHg for a minimum of 4 h prior to MRI. Vasopressors were allowed as feasible.
- Carbon dioxide levels between 30 and 40 mmHg for at least 4 h prior to MRI.
- Stable intracranial pressure for a minimum of 30 min and more in prone position with no elevation >25 mmHg. Use of osmotic agents was allowed as feasible.

Approval for MRI was given by the senior neurointensivist on duty.

### IHT and MRI Examination

Two staff members of the NICU, including one junior or a senior board-certified physician, performed the transport. Each patient was on ventilator support using an Oxylog 3000 (Dräger, Lübeck, Germany) and monitored by a DATEX Omeda Monitor (GE Datex Ohmeda, Helsinki, Finland). During the MRI examination, all patients were under anesthesiological supervision by a board-certified anesthetist. The patients were ventilated and monitored using an Aestiva 5 MRI, DATEX Omeda Monitor (GE, Germany). Standard analgosedation consisted of sufentanyl, propofol, and/or morphium plus midazolam. In some cases, ketanest was additionally administered. No patients received barbiturates. Medication and cardiopulmonary parameters were recorded at an interval of 15 min. Intracranial pressure was obtained at the NICU 1 h before the scheduled IHT, immediately before and after IHT, and 1 h afterward.

In the NICU and during IHT, the head was elevated 30° head-of-bed position. During the MRI scan, the patient was in a prone position with a 0° head-of-bed position, due to the standard spatial and technical circumstances. Repositioning on the MRI table was performed by at least four hospital staff members. Given the site characteristics, transporting the patient from the NICU to the MRI required a double floor change, i.e., taking the elevator twice.

### Magnetic Resonance Imaging

All patients underwent standard MRI including the following sequences: DWI, FLAIR, 3D T2-space, 3D T1 MPRAGE, 3D SWI, 3D TOF, and pASL. MRI was performed on a 3T whole-body system (Verio, Siemens Medical AG, Erlangen, Germany). MR spectroscopy was performed on the same MRI unit with a double-tuned 1H/31P volume head coil (Rapid Biomedical, Würzburg, Germany). For each patient, a scan time of 60 min was planned, not including the time of repositioning the patient and change of the head coils.

### Statistical Analysis

Statistical analysis was conducted using IBM SPSS Statistics (V.21, version 21, SPSS Inc., IBM, Chicago, IL, USA). Continuous variables were reported as mean ± standard deviation (SD). To detect differences between time points, the pairwise Wilcoxon test was used. Differences with a value of *p* < 0.05 were considered statistically significant. Graphs were created using GraphPad Prism (version 6.0c; for Mac; GraphPad Software, La Jolla California USA).

## Results

### Patients

A total of 26 patients (aged 23–78 years, mean 46.4 years; four female and 22 male patients) with severe TBI were enrolled in the analysis. Median GCS on admission was five (range 3–8).

### Imaging

Sixty-five MRI examinations were scheduled, 19 (29.2%) were not pursued due to the following reasons: three patients (4.6%) died prior to the first MRI, and in 16 patients (24.6%) MRI slots and/or any anesthesia teams were not available. In total, the pre-MRI test battery was performed 46 times. Of these, six patients (13.0%) failed due to increased ICP, and therefore, no MRI was performed. Finally, 40 IHT for MRI were performed within the first 14 days after the trauma with a mean of 6 days (range 1–14). A single MRI examination was performed in 13 patients, two MRI scans in 12 patients, and three MRI examinations in one patient. One patient passed the test, but showed an increase in the ICP prior to the MRI, which was therefore canceled (2.2%).

Mean duration of the MRI scan including positioning the patient on the MRI table and installing the monitoring was 82 min (range 45–120 min).

### Intracranial Pressure

**Figure 1:**
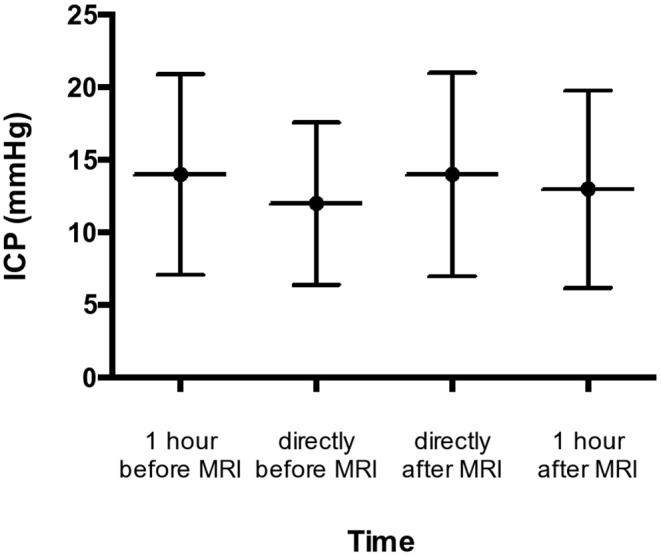
Pre- and post-examination ICP courses.

Mean pre-examination ICP ranged from 3 to 32 mmHg 1 h before the scheduled MRI (mean: 14.1 mmHg) and remained stable ranging between 3 and 25 mmHg (mean 12.8 mmHg) directly before the IHT to the examination. Being back at the NICU, the mean ICP was 14.3 mmHg (range 3 to 29 mmHg), slightly decreasing to a mean ICP of 13.2 mmHg after 1 h (range 3 to 29 mmHg). Values above 25 mmHg were seen 1 h before MRI and after MRI in two patients, respectively. There were no significant differences between the measurements in any time point during the MRI examination (Figure 1). The canceling of anticipated MRI was necessary only in one patient who passed the test battery, yet developed an increased ICP shortly before imaging. Noteworthy, the same patient underwent testing again the other day, passed the test battery, and MRI was performed without any issues.

### Cardiopulmonary Parameters

**Figure 2:**
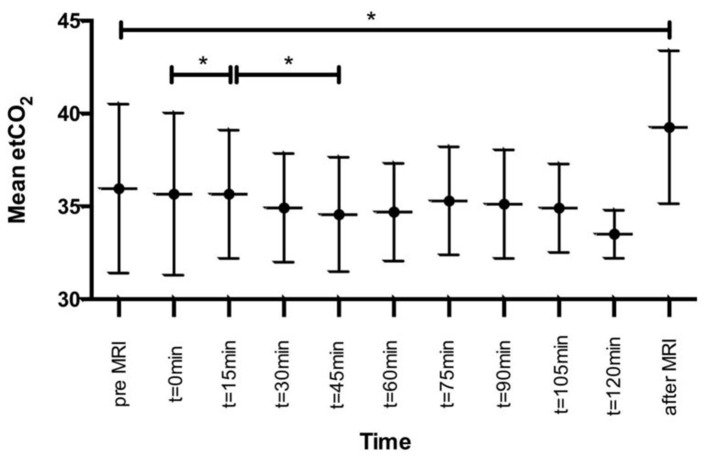
Pre- and post-examination etCO_2_ courses. *statistically significant.

Pressure-controlled ventilation was used in 36 patients (90%) and volume-controlled ventilation in four cases (10%). MAP remained stable throughout the entire course with no significant changes. EtCO_2_ differed before, during, and after the MRI examination, yet clinically not relevant. All data are listed in Table 1 and Figure 2.

**Table 1:** Pre- and post-examination MAP and etCO_2_ courses.

	**EtCO_**2**_**	**MAP**
Pre transport (NICU)	36.0 (27–47)	90.6 (70–119)
Start MRI (0min)	36.7 (27–46)	91.3 (72–113)
After 15min	35.6 (29–44)	90.9 (73–112)
After 30min	34.9 (27–40)	90.6 (73–113)
After 45min	34.6 (27–40)	91.0 (73–110)
After 60min	34.7 (30–40)	91.4 (73–113)
After 75min	35.3 (30–40)	92.3(70–113)
After 90min	35.1 (30–39)	95.4(73–112)
After 105min	34.9 (32–38)	95.4(78–110)
After 120 min	33.5 (32–35)	92.1(77–105)
Post transport (NICU)	39.3 (33–48)	90.6 (50–140)

### Medication

Neosynephrine was administered during 38 intrahospital transports/MRI examinations, in a concentration of 10 mg/50 ml in 31 cases and of 50 mg/50 ml in seven patients. Dobutamine was applied in one patient (concentration 250 mg/50 ml). Eight patients required no catecholamine support.

The utilization of sedatives and analgesics is listed in Table 2. The most commonly used analgesic was sufentanyl (85%), and the most common sedative medication was midazolam (92.5%).

**Table 2:** Utilization of sedatives and analgetics.

**Combination**	**Number**	**Percentage**
Midazolam + Sufentanyl + Ketanest	16	40%
Midazolam + Sufentanyl	18	45%
Midazolam + Vendal	3	7.5 %
Propofol alone	3	7.5 %

Additionally, procuronium was administered during 18 MRI examinations (ranging from 30 to 100 mg adapted to patient's body weight). Ephedrine was administered as a bolus to maintain/optimize MAP in two examinations.

## Discussion

Our study of sTBI patients who received MRI within 14 days after trauma provides representative comprehensive data on the feasibility and safety of MRI in the acute phase after severe TBI.

Overall, we have demonstrated that with proper preparation and accurate patient selection, MRI is possible in the vast majority of these severely injured patients with neither significant changes in cardiac/pulmonary parameters nor ICP. To our best knowledge, this is the first clinical prospective study showing the safety and feasibility of early MRI examination in these patients.

Diffuse axonal injuries (DAI), frequently occurring after TBI often leads to cognitive and behavioral impairment. Structural neuroimaging methods are insensitive in detecting such alterations. Thus, there has been a rapidly increasing interest in MRI that can provide a more detailed analysis of all intracranial lesions, giving an objective assessment of the degree of diffuse tissue injury, microhemorrhages, and posttraumatic “tissue at risk.” This is normally seen in the pericontusional region, where diffuse tensor imaging (DTI) can detect areas with vasogenic or cytotoxic edema, while the area of decreased ADC represents edema. Prevention of contusion growth represents a potential therapeutic target in the prevention of secondary injury in TBI (11). MRI after TBI yields essential prognostic information and offers a high potential to determine the degree of injury, and to improve stratification, in order to identify patients who require an extended period of intubation and intensive care management and to provide guidance in early decision making (2, 3, 12–14).

The proper timing of MRI continues to be debated, as MRI in ventilated patients in an early stage is highly challenging and related to numerous critical care issues. Manolakaki et al. suggested that early MRI is of limited usefulness in patients with TBI since it hardly changed patient management in the acute phase (15). This can be debated as “tissue at risk” may well be identified. Nevertheless, MRI is seen as very useful in predicting short-term and long-term functional outcome (16, 17). Neurointensivists, nowadays, are confronted with the dilemma when to perform MRI in critically ill patients. It is known that transferring these patients from the NICU is associated with a variety of medical complications or technical equipment failure. In general, IHT is deemed hazardous in critically ill patients with common complications such as thrombosis, bleeding, or decline in pulmonary function (18). Bergman et al. also reported technical hazards and equipment failure (19). Similar to our study, Martin et al. focused on severe TBI patients. In their cohort of 31 patients, a significant risk for secondary insults and adverse events was demonstrated in IHT to perform computed tomography (CT) scan. However, all documented events may have resulted in a change in medical therapy, but did not significantly alter outcome (9). Similar findings have been reported by Kleffmann et al. who observed a significant ICP increase during transport and CT scans (7). Notably, in their series of 14 patients with TBI, no GCS was provided, rendering comparison to our cohort difficult. Picetti et al. also demonstrated a higher risk for intracranial hypertension during IHT, but their cutoff value of 20 mmHg was fairly low for sufficient comparison with our findings (8). Partially corresponding to our results, they reported an elevated etCO_2_ after the imaging (8). Likewise, we also consider this to be of little clinical significance as etCO_2_ values were substantially lower during the time of MRI examinations.

It is our firm belief that the very low rate of failed MRI (2.2%) in our study results from our standardized preparation for MRI including the test battery and consistent patient selection. Of note, Tobin et al. reported a rate of 87% successful MRI in critically ill children, among them 10% with TBI (20). Our aforementioned standard operating procedure with the careful patient selection is in line with other studies. Both Cuschieri et al. and Berkow et al. suggested an implementation of standardized operating procedures in critically ill patients to improve patient outcome and morbidity (21, 22). A recent review on IHT stated that proper stabilization of the patient before anticipated intervention prevents directly IHT-related adverse events or complications (23). Importantly, as previous reports dealing with MRI in critically ill patients described, coordination with the MR facility is mandatory to decrease delay and minimize the length of the IHT (24).

We acknowledge that our study has several limitations. Certainly, our results must be interpreted carefully in light of the small sample size, even though similar to other studies (5, 9). Also, given the possible adverse effects of ICP probes in surrounding brain tissue during the MRI examination, continuous measurements of intracranial pressure was not possible during MRI as the intraparenchymal probes have not been approved/eligible for this purpose (25). It is therefore possible that episodes of high intracranial pressure may have gone unnoticed. However, relevant intracranial hypertension during MRI would have very likely provoked accompanying significant changes in other recorded parameters, or even ICP decompensation immediately after imaging, which was not the case in any of the included patients. Additionally, our analysis focused primarily neither on complications during IHT directly nor on therapeutic decision changes due to findings in the MRI.

To the best of our knowledge, this is the largest series regarding early MRI in ventilated patients with severe TBI. Despite the very challenging character of early MRI in critically ill patients and possible technical difficulties associated with required MR compatibility, we have demonstrated that early magnetic resonance after severe TBI is technically feasible, representing a promising non-invasive tool for comprehensive assessment of brain damage and posttraumatic “tissue at risk,” with an obvious potential to provide better guidance of clinical therapy and prediction of overall neurological outcome. Proper testing of relevant clinical parameters confirming the stable clinical condition of patients in a standardized manner plays a crucial role in avoiding related complications in these patients. Furthermore, IHT of critically ill and/or ventilated patients should not limit further meaningful clinical trials in these patients that are indisputable for better decision making in a daily clinical practice.

## Conclusion

Early MRI is feasible and safe in acute severe TBI when patients are carefully selected and properly tested in a standardized manner. To minimize MRI- and IHT-related risks and complications in these patients, consistent intensive monitoring and management of critically ill patients should be performed in close cooperation with anesthesiologists and neurointensivists.

## Data Availability Statement

The datasets used and/or analyzed during the current study are available from the corresponding author on reasonable request.

## Ethics Statement

The study protocol was approved by the local ethics committee of the Medical University Innsbruck (AN2014-0201 339/4.6).

### Consent for Publication

Written informed consent was obtained from all included patients and/or from their legal guardians.

## Author Contributions

DP: acquisition, analysis of data, and interpretation of data. ML: acquisition and interpretation of data. IB and MB: acquisition and analysis of data. CT: design of the study and revisions. OP: conception/design of the study, acquisition, analysis of data, and interpretation of data. All authors have approved the submitted version and have agreed both to be personally accountable for the author's own contributions and to ensure that questions related to the accuracy or integrity of any part of the work, even ones in which the author was not personally involved, have been appropriately investigated, resolved, and the resolution documented in the literature.

### Conflict of Interest

The authors declare that the research was conducted in the absence of any commercial or financial relationships that could be construed as a potential conflict of interest.
